# Misdiagnosis of Acute Appendicitis Cases in the Emergency Room

**DOI:** 10.7759/cureus.57141

**Published:** 2024-03-28

**Authors:** Reham Mostafa, Khaled El-Atawi

**Affiliations:** 1 Department of Emergency Medicine, Al Zahra Hospital Dubai (AZHD), Dubai, ARE; 2 Pediatrics/Neonatal Intensive Care Unit, Latifa Women and Children Hospital, Dubai, ARE

**Keywords:** imaging technique, diagnostic approach, emergency department, misdiagnosis, acute appendicitis

## Abstract

Acute appendicitis (AA) is one of the most frequent surgical emergencies, especially in pediatric populations, with its misdiagnosis in emergency settings presenting significant health risks. This misdiagnosis leads to various complications, such as delayed treatment or unnecessary surgeries. Factors such as age, gender, and comorbidities contribute to diagnostic errors, leading to complications such as peritonitis and increased negative appendectomy rates. This underscores the importance of accurate clinical assessment and awareness of common pitfalls, such as cognitive biases and over-reliance on laboratory tests. This review delves into the prevalence of AA misdiagnosis, its health burden, and the challenges inherent in the diagnostic process. It scrutinizes the effectiveness of different diagnostic approaches, including clinical assessment and imaging techniques. The treatment paradigms for AA are also explored, focusing on surgical interventions and the potential of conservative treatments using antibiotics. The review underscores the criticality of precise diagnosis in preventing adverse outcomes and ensuring effective treatment.

## Introduction and background

Abdominal pain is a leading reason for visits to the emergency department (ED) among children. In the United Kingdom, abdominal pain was found to be attributed to over 1,312,000 visits annually by those under 15 years old [1]. While most of these cases are not serious, identifying those needing additional investigation, imaging, or surgery is critical. Acute appendicitis (AA) is the top surgical emergency in this age group, leading to 72,000 hospitalizations each year [2]. However, diagnosing AA in pediatric patients is a complex task. This complexity is due to non-typical symptom presentations [3,4] and the challenges in obtaining reliable histories and physical examinations, particularly in younger patients [5,6]. Early detection of AA is essential to avoid severe outcomes such as perforation and abscesses [7,8].

The ED is a hotspot for diagnostic inaccuracies [9,10]. It is estimated that around 80% of these errors could be prevented [11], and nearly half of all diagnostic errors may harm patients [12]. Highlighting the gravity of this issue, the National Academies of Sciences, Engineering, and Medicine have declared enhancing diagnostic processes a critical public health goal [11,12]. Various methodologies have been adopted to scrutinize diagnostic errors [13]. Among these, a recently introduced conceptual framework employs a symptom-disease pairing for analytical purposes [14]. This method is particularly effective when a direct biological link exists between one or multiple symptoms and the resulting illness. In practice, this implies that the symptoms should have guided the healthcare provider toward a prompt and correct diagnosis [14]. Using symptom-disease dyad analysis on extensive datasets, including claims data, has been instrumental in pinpointing probable diagnostic oversights. This approach has been employed to approximate the frequency of overlooked diagnoses in various scenarios, such as chest pain not identified as myocardial infarction [15], misinterpreted dizziness or headaches leading to missed stroke diagnoses [16], and failure to recognize oncology-related symptoms in pediatric cancer cases [17]. These analyses provide a broader understanding of the prevalence of diagnostic errors at the population level. In this review, we shed light on the prevalence and consequences of misdiagnosis of AA, the factors associated with misdiagnosis of AA, the differential diagnosis of AA, and the potential strategies to avoid misdiagnosis of AA.

## Review

Epidemiology of AA and prevalence of misdiagnosis of AA

AA is more prevalent in males, with the male-to-female incidence ratio being roughly 1.4:1. Males have an 8.6% lifetime risk of developing AA compared to 6.7% for females [18,19]. Interestingly, the lifetime risk of undergoing an appendectomy is higher for females (23.1%) than males (12%). This higher rate of appendectomies in females is attributed to a greater likelihood of incorrect diagnoses, often due to female-specific pelvic issues that can be mistaken for AA [19].

In terms of legal implications, AA ranks as the second most common cause of malpractice claims among children aged 6-17 years, the third most common for patients aged over 18 years, and the most frequent in abdominal pain-related litigation. About 10% of all closed malpractice claims are related to missed AA cases [20,21]. The disease is less common in individuals under 5 or over 50 years old. In these age groups, AA often presents with atypical symptoms, leading to a higher likelihood of delayed diagnosis and complications such as ruptured appendices. The mortality rate from AA is usually below 1% but can escalate to between 5% and 15% in elderly patients [22,23]. AA is believed, though not conclusively proven, to have both genetic and environmental factors [24]. This theory is supported by a small case-controlled trial and a retrospective twin study from Australia, suggesting a genetic link to the disease [24,25].

Risk factors identified for AA include being male, Caucasian, and presenting symptoms during the summer. The disease is most common in younger individuals, with 69% of cases in those under 30 years old, peaking in males aged 10-14 years and females aged 15-19 years. Some studies suggest that low dietary fiber intake might increase the risk of AA, although these studies suffer from limitations such as small sample sizes or recall bias [26,27]. Additionally, there is some evidence linking tobacco use to an increased risk of AA [28,29].

The prevalence of AA misdiagnosis was investigated in a few studies. Weinberger et al. conducted a retrospective study of 1,378 patients who underwent appendectomy or were diagnosed with periappendicular abscess [30]. Their findings showed that in 7.1% of these cases, the diagnosis of AA was missed during their first visit to the ED, and they were either discharged home or hospitalized in a department other than general surgery. Mahajan et al. included 187,461 patients with a diagnosis of AA. They showed that the diagnosis of AA was missed in 6.0% of the adults and 4.4% of the children [31].

Factors associated with misdiagnosis of AA

In the Mahajan et al. study, the authors reported several factors associated with the misdiagnosis of AA. First, they found a significant difference between adult patients diagnosed on the same day and those with misdiagnosis in terms of age, sex, ethnicity, comorbidity index, and laboratory tests. Patients who were misdiagnosed were associated with older age, higher comorbidity index, and higher proportions of female sex and white ethnicity. Similarly, in children, misdiagnosed patients were commonly females with higher comorbidity index. However, there was no significant difference in terms of age or ethnicity. Regarding the presentation, patients who presented with abdominal pain only, abdominal pain with nausea and vomiting, and abdominal pain with nausea and vomiting and fever or constipation were associated with less likelihood of misdiagnosis. On the other hand, patients who presented with abdominal pain and constipation and those without abdominal pain were associated with a higher risk of misdiagnosis. Among children, patients who presented with abdominal pain and were evaluated using radiological modalities alone or complete blood count alone were associated with a significantly higher risk of misdiagnosis [31]. According to Weinberger et al., female sex was associated with a significantly higher risk of hospitalization due to AA misdiagnosis. Additionally, they found that patients who presented with >24 hours of symptoms before the first ED presentation were associated with a higher risk of misdiagnosis [30]. It was found that AA misdiagnosis is frequent in children presenting with constipation [32]. The presence of constipation during the initial visit to the ED might influence the clinical decisions of ED physicians, potentially increasing the likelihood of false-negative results for AA. In contrast, there is a lack of similar data for adults with a missed appendicitis diagnosis. The presence of constipation may prompt practitioners to opt for radiography, which could lead to a confirmation bias in their initial diagnosis and a premature conclusion in the diagnostic process despite the uncertain efficacy of radiography in diagnosing constipation [33,34].

Consequences of misdiagnosis

The misdiagnosis of AA can have profound and sometimes life-threatening consequences. When AA is not correctly identified, there is a significant risk of the appendix rupturing, leading to peritonitis, a severe and potentially fatal inflammation of the abdominal lining [35]. This complication necessitates more extensive surgical intervention and can result in prolonged hospital stays, increased healthcare costs, and a higher risk of postoperative complications. In pediatric patients, the consequences are particularly critical, as delayed treatment can lead to more severe outcomes, including systemic infections. Additionally, misdiagnosis can result in unnecessary surgical procedures, such as negative appendectomies, where patients undergo surgery without having actual appendicitis. These unnecessary surgeries not only expose patients to the risks associated with any surgical intervention, including infections and anesthesia-related complications but also contribute to increased healthcare expenditures and resource utilization [36].

Common pitfalls in clinical assessment

Clinical assessment of acute conditions such as AA is fraught with challenges that can lead to diagnostic pitfalls. One of the primary issues is the variability of symptom presentation, particularly in pediatric and elderly populations. In children, symptoms can be atypical, and communication barriers can hinder accurate symptom descriptions, leading to misinterpretation. In the elderly, the blunted immune response and atypical presentations can easily mask the severity of the condition. Another common pitfall is the over-reliance on laboratory tests and imaging results. While these tools are invaluable, they are not infallible and can lead to false negatives or false positives. This over-reliance can overshadow clinical judgment, which is crucial in assessing the likelihood of AA.

Additionally, cognitive biases such as confirmation bias and anchoring bias play a significant role in misdiagnosis. Healthcare providers might focus on initial impressions or the most prominent symptoms, leading to early closure in the diagnostic process without considering alternative diagnoses. This issue is compounded by the high-pressure, time-constrained environment of emergency settings, where rapid decision-making is often required. Furthermore, a lack of experience or familiarity with the wide range of AA presentations can result in inadequate physical examinations and history taking. These pitfalls underscore the need for comprehensive, systematic approaches in clinical assessments and continuous education and training for healthcare providers to improve diagnostic accuracy.

Diagnostic approaches to avoid AA misdiagnosis

Clinical Presentation

The diagnostic process for AA, often described as the “chameleon of surgery” [37], is complicated due to its highly variable clinical presentations and numerous differential diagnoses. Especially in children, where up to 50% of cases manifest with non-specific symptoms, diagnosing appendicitis can be challenging [38,39]. It is advisable to categorize appendicitis as either uncomplicated or complicated before starting treatment to ensure appropriate care. Key aspects of patient history, such as symptom onset, pain location, past medical history, and current medications (Table 1), are critical in diagnosis. A classic sign of appendicitis is the migration of pain from the upper abdomen to the right lower quadrant [40]. For children and adolescents, tailoring the history and physical examination to their age and developmental level is essential, and the examiner’s experience plays a vital role, especially with younger children. Administering analgesics does not significantly obscure clinical findings [41]. In pediatric cases, the absence of symptoms such as nausea, vomiting, abdominal tenderness, and leukocytosis effectively rules out appendicitis with a 98% accuracy [42].

**Table 1:** Overview of criteria for uncomplicated versus complicated appendicitis, adapted from those of the European Association of Endoscopic Surgery (EAES) [43] and of diagnostic measures in suspected acute appendicitis. Complicated appendicitis is present, by definition, if any criterion in addition to inflammation is met. + = yes; - = no; ± = possible; * = in female patients of child-bearing age; ** = method of first choice

Domain	Uncomplicated	Complicated
Criteria for uncomplicated versus complicated appendicitis		
Inflammation	+	+
Gangrene	−	+
Phlegmon	−	+
Perityphlitic abscess	−	+
Free fluid	−	+
Perforation	−	+
Diagnostic measures		
History	+	+
Physical examination, including appendicitis pressure points	+	+
Digital rectal examination	−	−
Laboratory tests	+	+
Body temperature measurement	+	+
Urine test strip and pregnancy test*	+	+
Gynecological consultation	±	±
Abdominal ultrasonography**	+	+
Computed tomography	−	±
Magnetic resonance imaging	−	±

Scoring System

Numerous scoring systems have been established to assist in the objective assessment and confirmation of AA, independent of the examiner’s clinical experience. The Alvarado score, introduced in 1986, and the Appendicitis Inflammatory Response (AIR) score from 2008 are among the most frequently utilized (Table 2) [44]. The Alvarado score is highly sensitive (99%) when the score is equal to or greater than 5, but it has a relatively low specificity of 43%. Increasing the threshold to 7 or more enhances specificity to 81%, though this reduces sensitivity to 82%. Consequently, the Alvarado score is more effective in excluding appendicitis than in confirming it. On the other hand, an AIR score exceeding 8 showcases high sensitivity and specificity (99%) in diagnosing appendicitis [44,45]. Despite the utility of these scoring systems, they are not widely adopted in routine clinical diagnostics in Germany. Another scoring system is the Adult Appendicitis Score (AAS), which stratifies patients into three risk groups based on their scores. A score of 16 or higher indicates a high risk of appendicitis, 11-15 indicates an intermediate risk, and 10 or less indicates a low risk [46].

**Table 2:** Modified summary of the Alvarado [47] and AIR scores [48] for evaluating the possible presence of appendicitis. AIR = Appendicitis Inflammatory Response; CRP = C-reactive protein; RLQ = right lower abdominal quadrant; PMN = polymorphonuclear

Criterion		Alvarado score	AIR score
Symptoms			
Vomiting		–	1
Nausea or vomiting		1	–
Anorexia		1	–
RLQ pain		2	1
Migratory RLQ pain		1	–
Signs			
RLQ rebound pain or guarding	Overall	1	–
	mild	–	1
	moderate	–	2
	severe	–	3
Body temperature	>37.5°C	1	–
	>38.5°C	–	1
Laboratory parameters			
Leukocyte count	>10,000/L	2	–
	10,000–14,900/L	–	1
	>15,000/L	–	2
Leukocyte shift	Leukocyte shift	1	–
PMN granulocytes	70–84%	–	1
	≥85%	–	2
CRP value	10–49 mg/L	–	1
	≥50 mg/L	–	2
Sum		10	12
Alvarado score		<5	Low probability
		5–6	Unclear
		7–8	Likely
		>8	High probability
AIR score		<5	Low probability
		5–8	Intermediate probability
		>8	High probability

Imaging

In evaluating suspected AA, ultrasonography, abdominal CT, and MRI are all employed, with ultrasonography being the preferred method, especially for pediatric cases [49]. The effectiveness of ultrasonography largely depends on the examiner’s experience, and a negative result does not definitively exclude appendicitis, with its sensitivity ranging between 71% and 94% and specificity between 81% and 98% [43,45]. In children, ultrasonography shows higher sensitivity (96%, 95% confidence interval (CI) = 83% to 99%) and specificity (100%, 95% CI = 87% to 100%) [49]. Abdominal CT scans offer better sensitivity (76%-100%) and specificity (83%-100%) compared to ultrasonography [43]. However, the use of CT in AA is debated in Western countries and varies by region. In the United States, CT scans are commonly used in 72%-95% of cases, contributing to the lower rate of negative appendectomies (less than 5%) [50-52]. In contrast, European practices often rely on clinical diagnosis, leading to a higher frequency of laparoscopies and a greater incidence of negative appendectomies (up to 32%) [53,54]. Employing low-dose CT instead of conventional CT does not significantly change the rate of negative appendectomies (difference = 0.3%, 95% CI = -3.8 to 4.6), and omitting oral contrast while using intravenous contrast can reduce radiation exposure without compromising diagnostic sensitivity (95% versus 92%) [55-59]. However, CT, despite its high sensitivity and specificity, may struggle to differentiate between complicated and uncomplicated appendicitis. In obese individuals (body mass index >30 kg/m^2^), the ultrasonographic signs of appendicitis are challenging to interpret, leading to more frequent CT use in this group [43]. This is also true for individuals over 65 years of age, as they often present atypical symptoms and a wider range of differential diagnoses due to increased comorbidities. MRI has similar sensitivity (97% versus 76%-100%) and specificity (95% versus 83%-100%) to CT in diagnosing AA [43], but its availability in emergency settings is limited. MRI is considered a safer alternative to CT for children and pregnant women, as it does not involve ionizing radiation. It is preferred if ultrasonography results are inconclusive [60]. For children under five, sedation or general anesthesia is often required for an MRI.

Treatment

The World Society of Emergency Surgery (WSES), Society of Gastrointestinal and Endoscopic Surgeons (SAGES), and European Association for Endoscopic Surgery (EAES) all endorse appendectomy as the preferred treatment for uncomplicated AA across all age groups [43,45,61]. The concept of conservative treatment for AA was first introduced by Harrison in 1953 [62], and some early reports indicated cases of spontaneous resolution [63]. In recent times, the notion of treating uncomplicated appendicitis conservatively with antibiotics in both children and adults has gained traction, supported by numerous studies [43,64-66]. Recent meta-analysis data show that 92% (95% CI = 88 to 96) of children and adolescents with uncomplicated appendicitis treated non-surgically experienced symptom resolution, though 16% (10%-22%) eventually required an appendectomy due to recurrence within a follow-up period of 8 weeks to 4.5 years [67]. A 2016 meta-analysis found no significant difference in complications or hospital stay duration [68]. However, Kessler et al.’s comparative analysis indicated higher hospital readmission rates (relative risk (RR) = 6.98, 95% CI = 2.07 to 23.6) and a lower likelihood of symptom resolution (RR = 0.77, 95% CI = 0.71 to 0.84) with conservative management in children [69].

The studies in these analyses were non-randomized with smaller-than-ideal patient groups, suggesting that children with milder symptoms were more likely chosen for conservative treatment. Long-term follow-up studies revealed significant appendectomy rates post-initial conservative treatment: 46% in children after five years [70] and 27% and 39% in adults at one and five years, respectively, in the APPAC trial [71,72]. The APPAC trial also failed to meet the non-inferiority criterion for antibiotic treatment after one year. Despite these analyses, the evidence is still considered insufficient to recommend a shift in clinical approach, although conservative treatment is seen as safe [67-69]. A 2019 meta-analysis of five randomized controlled trials found appendectomy more effective for definitive treatment of uncomplicated appendicitis in adults, with a 37.4% appendectomy rate within a year after conservative treatment and a treatment effectiveness rate of 62.6% compared to 96.3% in the surgical group [73]. Current evidence does not show a clear advantage for conservative treatment, thus appendectomy remains the standard approach for treating acute uncomplicated appendicitis in both children and adults [67]. Future trials (APPAC-III, MAPPAC) are set to investigate the potential long-term adverse effects of conservative treatment, such as drug side effects and antibiotic resistance [74,75]. The APPAC III trial is a multicenter, double-blind, placebo-controlled study that aims to evaluate the role of antibiotics in the resolution of CT scan-confirmed uncomplicated acute appendicitis. The primary endpoint is the success of the randomized treatment, defined as the resolution of acute appendicitis resulting in discharge from the hospital without surgical intervention within 10 days after initiating randomized treatment [74]. The MAPPAC trial is a prospective clinical study aiming to evaluate the microbiological and immunological aspects of different forms of acute appendicitis, assess the response to antibiotics, and study the recurrence of appendicitis. The trial will also investigate the effects of antibiotics and placebo on gut microbiota composition and antimicrobial resistance, and it will recruit patients with uncomplicated and complicated appendicitis [75].

## Conclusions

The challenge of accurately diagnosing AA in emergency settings, particularly in pediatric patients, is critical. The significant risks associated with misdiagnosis, including severe complications and increased healthcare costs, underscore the need for improved diagnostic strategies. The variability in symptoms and the potential for cognitive biases in clinical assessments highlight the necessity of a systematic, multifaceted approach to diagnosis. Future directions should focus on advancing diagnostic tools and techniques, enhancing training for healthcare providers, and further investigating the effectiveness of conservative treatment methods. The integration of new technologies and continuous research, particularly in areas such as conservative treatment and its long-term effects, will be vital in evolving the clinical approach to AA.
